# The Legacy of Olympic Sport: A Cross-Sectional Comparison of the Musculoskeletal and General Health of 1488 Retired Female Summer and Winter Olympians and 998 Female General Population Controls

**DOI:** 10.1007/s40279-026-02497-2

**Published:** 2026-07-23

**Authors:** Lauren Guilfoyle, Alan MacPherson, Dale Cooper, Kay M. Crossley, Amber T. Donaldson, Niall Elliott, Lars Engebretsen, Suzanne Leclerc, Eric G. Post, Craig Ranson, Patrick Schamasch, Malav Shroff, Jenna M. Schulz, Torbjørn Soligard, Jane S. Thornton, Mike Wilkinson, Debbie Palmer

**Affiliations:** 1 Edinburgh Sports Medicine Research Centre, University of Edinburgh Institute for Sport Physical Education and Health Sciences, Edinburgh, UK; 2 Collaborating Centre on Injury and Illness Prevention in Sport, University of Edinburgh, Edinburgh, UK; 3 School of Allied Health Professions, Keele University, Keele, Staffordshire UK; 4 La Trobe Sport and Exercise Medicine Research Centre, La Trobe University, Melbourne, Australia; 5 Australian IOC Research Centre, La Trobe University, Melbourne, Australia; 6 Department of Sports Medicine, United States Olympic & Paralympic Committee, Colorado Springs, CO USA; 7 US Coalition for the Prevention of Illness and Injury in Sport, Colorado Springs, CO USA; 8 Department of Sports Medicine, Oslo Sports Trauma Research Center, Norwegian School of Sport Sciences, Oslo, Norway; 9 Department of Family Medicine, University of Montreal, Montreal, QC Canada; 10 ReFORM IOC Research Centre for Prevention of Injury and Protection of Athlete Health, Liège, Belgium; 11 Department of Orthopedics and Rehabilitation, University of Wisconsin-Madison, Madison, WI USA; 12 UK Sports Institute, Manchester, UK; 13 Medical Committee, World Olympians Association, Lausanne, Switzerland; 14 Department of Physical Therapy, Faculty of Medicine, University of British Columbia, Vancouver, Canada; 15 Health, Medicine and Science Department, International Olympic Committee, Lausanne, Switzerland; 16 Health and Wellbeing Department, Canadian Olympic Committee, Toronto, Canada; 17 Department of Sports Medicine, Faculty of Medicine, University of British Columbia, Vancouver, Canada

## Abstract

**Background:**

Little is known about the long-term health outcomes associated with sustained elite performance once female Olympians retire from their Olympic careers.

**Objective:**

The aim of this study was to determine and compare (1) musculoskeletal health, (2) general health and (3) current physical activity (PA) behaviours between a global sample of female retired Olympians and general population controls.

**Methods:**

A cross-sectional survey comprised four sections: (1) background information, (2) injury history, (3) current musculoskeletal health and (4) general health. A total of 1488 retired female Olympians and 998 female controls completed the survey. Poisson regression analysis was used to determine prevalence ratios (aPR) with 95% confidence intervals (CI) adjusted for age, body mass index (BMI) and/or previous significant injury or current persistent pain.

**Results:**

Adjusting for age, BMI and previous significant injury, more retired female Olympians reported stiffness, pain and/or osteoarthritis (OA) (aPR [95% CI]) in the cervical spine (stiffness 1.42 [1.08–1.86]; OA 1.85 [1.01–3.39]), lumbar spine (stiffness 1.41 [1.18–1.67], pain 1.41 [1.15–1.73]) and hip (OA 1.82 [1.08–3.06]) compared with controls. Stiffness, pain and OA at the knee were also higher for Olympians, but not when adjusting for prior injury. No differences were observed between groups for self-reported disease states, however the prevalence of most health conditions increased among Olympians following retirement, except for depression which decreased. Both groups rated their health-related quality of life (retired female Olympians: 88/100; Controls: 89/100) and mental health similarly, while the prevalence of participation in weekly sport (aPR: 0.75 [0.66–0.85]) and physical activity (aPR: 0.91 [0.87–0.95]) was slightly lower in retired Olympians when adjusted for age, BMI and current persistent musculoskeletal pain.

**Conclusions:**

Retired female Olympians report poorer musculoskeletal health than the general population, a difference only partly attributable to previous significant injury, which suggests elements of elite athletic exposure may underlie some of these outcomes. Primary and secondary prevention of shoulder, hip and knee injuries remains a priority with consideration to long-term outcomes. Future research should broaden our understanding of retired female athlete health by exploring the largely under-researched domains of cognitive, reproductive, breast and pelvic floor health.

**Supplementary Information:**

The online version contains supplementary material available at https://doi.org/10.1007/s40279-026-02497-2.

## Key Points

**Table:** 

The self-reported musculoskeletal health of retired female Olympians is poorer than female general population controls, with an increased prevalence of pain, stiffness and/or osteoarthritis at the shoulder, cervical spine, lumbar spine, hip and knee—only some of which can be explained by prior significant injury.
Retired female Olympians rate their health-related quality of life and score their mental health similarly to controls, raising suggestions of differing pain tolerance, learned coping mechanisms and/or increased awareness of the therapeutic use of exercise to manage their symptoms.
Retired female Olympians report differing—mostly lower—levels of engagement in sport, exercise and physical activity behaviours; however, analysis failed to identify persistent pain as a barrier.

## Introduction

A milestone moment for equality among female athletes in Olympic sport was achieved at the 2024 Paris Olympic Games when gender parity was realised for the first time through equal athlete representation [1]. This comes in an era of increased rates of female participation in Olympic sport, higher levels of female-focused sponsorships and year-on-year growth in viewing figures for women’s events [2]. Coupled with the introduction of progressive moves from sporting bodies designed to remove barriers to attainment [3, 4], these parallel developments will likely extend high-performance career durations for female athletes in future Olympic cycles. While much is known about the health status of retired male elite athletes [5–7], much less is known about retired female Olympians—where data are either missing or the available data has not been disaggregated adequately to provide a true and comprehensive picture. Consequently, the potential association of intensive and sustained sport participation with long-term health outcomes of retired elite female athletes is currently reliant on the data derived from their retired male counterparts [8, 9].

Extrapolating retired male athlete data to inform downstream healthcare is insufficient for retired female athletes. Female athletes have different injury profiles than their male counterparts with higher risk of anterior cruciate ligament tears [10], bone stress injuries [11] and concussion [12] which can affect long-term joint, bone and cognitive health [13–15]. Physiologically, females experience normal hormonal variation across their lifespan, with known implications for long-term musculoskeletal and cardiovascular health [16, 17]. Female athletes are also subject to sex and gender-specific sociocultural influences such as inequitable funding and resources, inferior access to experienced support staff, ill-fitting equipment and unbalanced load demands (physical, psychological and social) during their careers [18]. Combined, a multi-layered interplay of factors unique to the elite female athlete may influence their health in later life. These complexities cannot be considered without disaggregating and/or reporting female-only data, which is essential to protecting and promoting female athlete health both in-career and following retirement from elite participation [8, 9, 18].

Addressing this gap in long-term health outcomes is critical for female athletes, given indications in the extant literature that warrant further comprehensive and nuanced investigation. Our prior studies reveal that retired female Olympians report a greater prevalence of significant career injury, persistent pain and increased likelihood of cervical spine, lumbar spine and knee osteoarthritis (OA) compared with male Olympians [19–21]. Furthermore, our analyses also illustrate increased odds of shoulder, cervical spine and knee OA when compared with the female general population [20, 21]. These findings suggest a greater musculoskeletal health burden, with female Olympians also reporting significantly greater use of painkillers and non-steroidal anti-inflammatory drugs (NSAIDs) during their careers compared with male Olympians [19]. The continued use of such medications into retirement, in light of residual musculoskeletal complaints [20, 21], remains underexplored. This represents an important knowledge gap given the potential health consequences associated with long-term NSAID use for chronic pain management [45]. Understanding patterns of pain-relieving medication use may also serve as a useful proxy for the impact of pain on a person’s quality of life [67].

Research beyond the domain of musculoskeletal health is also limited in this population, with published meta-analyses across other pertinent aspects of health (mental, cognitive, cardiovascular) largely—and sometimes entirely—comprised of male-only cohorts [5, 6, 13, 22]. In retired Olympians, females reported higher rates of depression on retirement from sport compared with males [19], while retired female Great Britain (GB) Olympians self-reported lower cardiovascular and respiratory diagnoses, but a higher prevalence of any cancer, compared with the female general population [23]. Similarly, a lower prevalence of hypertension, high cholesterol and diabetes was reported by retired female international rugby players and rowers compared with the female general population in Canada [24]. The exploration of these health domains in highly powered international multi-sport cohorts is now warranted, including consideration of additional factors that may shape these long-term outcomes—in particular, modifiable lifestyle behaviours such as physical activity (PA) in retirement, smoking history and alcohol consumption.

Regular PA that meets or exceeds established guidelines confers multi-system benefits that include cardiovascular disease and all-cause mortality, site-specific cancers and cognitive health in the general population [25]. Thus, PA and exercise levels (or conversely, sedentary behaviour) in athletic retirement may act as a mediator or confounder to long-term health outcomes. Similarly, smoking tobacco use and alcohol consumption also interact with long-term health outcomes in the general population [26, 27]. Given the established associations between these lifestyle factors and long-term health, these behaviours should be explored in this population to determine whether elite female athletes engage in health-promoting behaviours in retirement; and to identify areas for future holistic support and long-term health promotion initiatives.

The present study aimed to expand on our prior evaluation of retired Olympian health by examining differences in the health of a sample of retired female Olympians compared with female general population controls. Specifically, the objectives were to compare (1) current musculoskeletal health; (2) general health measures including disease states, mental health status, medication (painkillers and NSAIDs), alcohol and tobacco use; and (3) current physical activity and exercise behaviours.

## Methods

A cross-sectional design was employed to collect self-reported data from retired female Olympians (hereafter referred to as *retired Olympians*) and female general population controls (hereafter referred to as *controls*). Methods for the study have been previously described [19–21]. Data collection occurred between April 2018 and June 2019 via an online questionnaire that was made available in eight languages (Chinese, English, French, German, Japanese, Korean, Russian and Spanish). Translation of the survey was conducted by professional medical translators and included back translation quality assurance processes. In addition, final translations were checked for both content validity and linguistic accuracy by native-speaking study authors. In the case of validated outcome measures, validated translations of tools were used. Ethical approval for this study was obtained from the Edinburgh Napier University Ethics Committee (SAS/00011). This manuscript was prepared in accordance with the Strengthening the Reporting of Observational Studies in Epidemiology (STROBE) Statement for cross-sectional studies [28].

Retired female Olympians were chosen as the cohort of interest as qualification for the Olympic Games provided a consistent criterion of high performance across a multi-sport sample and therefore facilitates wider generalisability of our results. A retired female Olympian was defined as any individual aged ≥ 16 years, who has competed in at least one Olympic Games (Summer and/or Winter), but who no longer intends to qualify for, or compete in any upcoming Summer or Winter Olympic Games. This also included retired Olympians who may still have been competing recreationally, nationally, or internationally in their chosen Olympic sport. Controls were any female, aged ≥ 16 years, who had not competed at a Summer and/or Winter Olympic Games.

### Recruitment

Retired Olympians were recruited through the World Olympians Association (WOA) (e.g., OLY database), International Olympic Committee (IOC) platforms (e.g., Athlete365) and National Olympian Associations and National Olympic Committees (NOC) [19]. The control group recruitment strategy had three strands: (1) a ‘buddy’ system whereby participating retired Olympians recruited a similar age, same sex, non-Olympian friend or associate; (2) promotion through WOA and IOC communication channels and (3) promotion by the research group at academic and industry organisations and/or at local regional public leisure, medical and community centres [20, 21].

A participant information sheet provided detailed study information, including confidentiality and data handling. Participants were notified they were consenting to their anonymous data being used in the study through the completion and submission of the survey [19].

### Survey

Data were collected via an online web-based survey hosted by SurveyMonkey. Access to the retired Olympian survey was password protected, while the general population control survey was openly accessible [20, 21]. The survey comprised four sections: (1) background information, (2) sport participation and significant injury history, (3) current musculoskeletal health and (4) general health, quality of life, current weekly sport, exercise and leisure PA.

A detailed overview of the survey (item content, response format, origin, validity, reliability, differences between surveys etc.) is available in Supplementary Appendix 1, found in the electronic supplementary material [ESM]). Baseline questions included age, sex, ethnicity, country of residence, current height (cm) and weight (kg) to calculate body mass index (BMI, kg/m^2^) and education and employment status. Additionally, retired Olympians were asked to report their Olympic career height (cm) and weight (kg) to determine Olympic career BMI.

All participants were asked to recall all significant injuries that occurred at any time across their lifespan up to and including current injuries. Significant injuries were defined as any injury that caused pain and discomfort for most days for at least one month—an operational definition employed in earlier comparable research [19, 29]. Injury detail included injury location, injury type and injury cause [30]. Retired Olympians were also asked to describe their international career duration at senior level and their age at Olympic career retirement.

Current musculoskeletal joint health was ascertained with the question, “Do you currently experience pain, for most days of the last month, in this joint?”. This question was selected for consistency with a prior study of retired Olympians [29], where it was adapted from earlier osteoarthritis research [31]. A modified version of this question was also included to capture joint stiffness (“Do you currently experience stiffness, for most days of the last month, in this joint?”). Self-reported physician-diagnosed OA was determined by asking “Have you ever been diagnosed with OA in any of your joints by a medical professional?” [29]. Participants also reported their history of joint surgery, including surgery type (e.g., reconstruction or joint replacement).

For general health status, participants reported current disease states (in response to a predefined list developed de novo by the research steering group), current regular medication use, alcohol consumption (frequency and quantity in units) and tobacco use (cigarette, cigar or pipe). Additionally, retired Olympians were asked to report disease states during their careers from a predefined list. Anxiety and depression were captured using the Hospital Anxiety and Depression Scale (HADS) originally developed for use in the general population [32]. The HADS tool compares consistently with the Diagnostic and Statistical Manual of Mental Disorders (DSM-5) criteria relative to sensitivity and specificity [33]. This 14-item questionnaire employs a four-point Likert scale. Each subscale (anxiety and depression) has a maximum score of 21, with higher scores indicating more severe symptoms [32]. A cut-off score of ≥ 11 (sensitivity: 0.64, specificity 0.87 [33]) was adopted for both sub-scales of the HADS to identify cases of possible or probable depression and anxiety and to minimise false positives [34, 35].

Quality of life was measured with the EQ-5D-5L—a valid and reliable generic instrument describing health status in general populations [36]. It comprises two components: the descriptive system relating to five health dimensions (mobility, self-care, usual activities, pain/discomfort and anxiety/depression) and the visual analogue scale (VAS) rating [37]. Participants were asked to indicate their health state for each dimension by selecting the appropriate label response (no problems, slight problems, moderate problems, severe problems, unable to/extreme problems). Finally, they were asked to quantify their perception of overall health using a 100-mm VAS ranging from 0 to 100 [38]. For analysis, categorical responses were collapsed into ‘no problems’ and ‘any problems’.

Participants also reported their current typical weekly sport, exercise and leisure PA through pragmatic de novo questions. These questions specifically asked the participant to report the intensity (exercise) and hours per week (sport, exercise, leisure PA) in separate sport, exercise and physical activity sections. For retired Olympians, these questions included both Olympic career sport-related and post-Olympic career sport-related exposure. Sport was operationally defined as any organised competitive sport, while exercise was defined to participants through illustrative examples (e.g. running, swimming, aerobics, gym) as was leisure PA (e.g. walking, shopping, gardening, DIY).

### Patients and Public Involvement

Face validity of the questionnaire content and design was provided by an advisory group composed of nine retired Olympians (male and female) [19–21]. Amendments to the final survey were made based on their feedback.

### Statistical Analysis

All analyses were conducted using Stata 19 software (StataCorp LLC, TX, USA). Descriptive statistics are presented as frequencies (proportion) for categorical variables and mean and standard deviation (SD), or median and interquartile range (IQR) where data are not normally distributed, for numerical variables. Six joints/anatomical regions (shoulder, cervical spine, lumbar spine, hip, knee and ankle) were selected a priori for further detailed analysis as these regions were most commonly affected across both groups [20, 21]. Prevalence was calculated by dividing the number of participants with the outcome of interest (e.g., injury, pain, stiffness) by the total number of participants in each group (Retired Olympians or controls) and presented as percentage (%) with 95% confidence intervals (95% CI). Poisson regression with robust standard errors was used to analyse prevalence ratios (PR) between groups and are presented with 95% Wald confidence intervals (CIs). The control group was consistently employed as the reference group. Adjusted prevalence ratios (aPRs) are presented, controlling for age and BMI in all instances, and additionally controlling for previous significant injury (yes/no) or current pain (yes/no) where specifically indicated. Differences between groups for continuous variables were analysed using independent t-tests or Mann–Whitney *U* tests. Internal consistency of the HADS tool was established using McDonald’s *ω* [39]. Quantile (median) regression—also adjusted for age, BMI and current pain (yes/no)—was used to compute adjusted median differences (aMD) in sport, exercise and leisure PA durations given these were not normally distributed. Statistical significance was set at *p* ≤ 0.05 and imputation was not undertaken for missing values. Where a total score is computed for validated tools, only complete responses (i.e., responses with no missing values) were included in analysis. Percentages are rounded to one decimal place.

## Results

Complete questionnaires were returned by 1488 retired female Olympians and 998 female general population controls (controls) (Supplementary Appendix 2, ESM). Retired Olympians represented a total of 132 NOCs at the Olympic Games (Supplementary Appendix 3, ESM), and 85% participated at the Summer Games (Supplementary Appendix 5, ESM). The median age was 41.3 years (range 16–88) and 40.6 years (range 16–87) for retired Olympians and controls, respectively. The ethnicity of both groups was predominantly white (retired Olympians: 78.1% versus controls: 90.0%). Demographic characteristics of both groups are presented in Table 1 with additional data presented in Supplementary Appendices 3–7 (see ESM).

**Table 1:** Retired female Olympian and female general population control characteristics

	Retired Olympians (*n* = 1488)	Controls (*n* = 998)	*p* value
Age [y], median (range)	41.3 (16–88)	40.6 (16–87)	0.003
Height [cm], mean (SD)			
Current	169.3 (8.6)^a^	167.2 (7.2)	< 0.001
Olympic career	169.5 (8.8)^a^	–	–
Weight [kg], mean (SD)			
Current	67.7 (14.3)^a^	68.4 (17.4)	0.334
Olympic career	63.0 (12.8)^a^	–	–
BMI [kg/m^2^], mean (SD)			
Current	23.5 (4.1)^a^	24.0 (4.4)	0.001
Olympic career	21.8 (3.3)^a^	–	–
Olympic sport, *n* (%)			
Summer	1264 (85.0)	–	–
Winter	224 (15.0)	–	–
International seasons [y], mean (SD)	9.9 (5.1)	–	–
Age at retirement from Olympic career [y], mean (SD)	28.2 (5.6)	–	–
Time since retirement from Olympic career [y], mean (SD)	15.8 (13.3)	–	–
Education, *n* (%)			
Did not finish school	7 (0.5)	1 (0.1)	0.149
Second-level^b^	336 (22.6)	197 (19.9)	0.102
Higher education^c^	1142 (76.9)	794 (80.0)	0.060
Current occupational status, n (%)			
Retired^d^	127 (10.4)	77 (9.0)	0.312
For health reasons	8 (0.6)	3 (0.4)	0.359
Unemployed	62 (5.1)	29 (3.4)	0.071
For health reasons	3 (0.3)	3 (0.4)	0.658
Employed	1034 (84.6)	746 (87.6)	0.049
Moderately physically demanding	172 (14.1)	89 (10.5)	0.015
Strenuous activity	45 (3.7)	19 (2.2)	0.064

*BMI* body mass index, *cm* centimetres, *SD* standard deviation

^a^Significant within-group differences (career vs retirement, where* p* ≤ 0.05)

^b^School or college

^c^Under-graduate or post-graduate

^d^Retired from employment

### Significant Injury History and Current Musculoskeletal Joint Health

A total of 1116 retired Olympians reported at least one significant Olympic career injury, equating to 1.4 injuries (95% CI 1.37–1.50) per Olympian and representing 75% of the sample (Table 2). More retired Olympians reported significant injury (79.1% vs 65.4%; aPR 1.22, 95% CI 1.15–1.29), across their life, compared with controls. Overall OA diagnoses, history of joint surgery and current joint stiffness (any joint) were also more prevalent among retired Olympians.

**Table 2:** Prevalence of significant injury and current musculoskeletal health in retired female Olympians versus female general population controls

	Retired Olympians (*n* = 1488)	Controls (*n* = 998)	Adjusted PR^a^ [95% CI]	Adjusted PR^b^ [95% CI]
Injury prevalence, *n* (%)				
Olympic career injury	1116 (75.0)	–	–	–
Non-Olympic career injury	408 (27.4)	653 (65.4)	**0.42 [0.38–0.46]**	–
All significant injury	1177 (79.1)	653 (65.4)	**1.22 [1.15–1.29]**	–
Total injuries, *n* (per individual)				
Olympic career injury	2133 (1.4)	–	–	–
Non-Olympic career injury	595 (0.4)	1376 (1.4)	**0.29 [0.25–0.32]**	–
All significant injury	2728 (1.8)	1376 (1.4)	**1.35 [1.24–1.46]**	–
Current injury (any joint), *n* (%)	292 (19.6)	139 (13.9)	**1.42 [1.17–1.73]**	–
History of joint surgery, *n* (%)	361 (24.3)	153 (15.3)	**1.56 [1.30–1.86]**	**1.31 [1.10–1.55]**
Current pain (any joint), *n* (%)	693 (46.6)	387 (38.8)	**1.23 [1.12–1.36]**	1.08 [0.98–1.18]
Current stiffness (any joint), *n* (%)	828 (55.7)	456 (45.7)	**1.25 [1.15–1.36]**	**1.10 [1.02–1.19]**
Current OA diagnosis (any joint), *n* (%)	303 (20.4)	131 (13.1)	**1.51 [1.24–1.84]**	**1.32 [1.10–1.60]**

Bold text indicates statistically significant

a*PR* adjusted prevalence ratio, *CI* confidence interval, *OA* osteoarthritis

^a^Adjusted for age and BMI

^b^Adjusted for age, BMI and previous significant injury in any joint (yes/no)

Prevalence and aPR of significant joint-specific injury history and previous surgical intervention are presented in Table 3. Prevalence of significant injury was highest in retired Olympians at the knee (26.8%), lumbar spine (21.2%) and shoulder (19.6%)—the prevalence of each being greater than controls (knee aPR 1.24, 95% CI 1.06–1.44; lumbar spine aPR 1.66, 95% CI 1.36–2.03; and shoulder aPR 1.59, 95% CI 1.29–1.94).

**Table 3:** Prevalence of significant injury and surgical intervention by joint, in retired female Olympians versus female general population controls

	Injury history		History of surgery		
	Injured participants *n* (%)	Injuries *n* (per participant)	Any surgery *n* (%)	Replacement *n* (%)	Reconstruction *n* (%)
Shoulder					
Retired Olympians	292 (19.6)	344 (0.23)	86 (5.8)	2 (0.1)	26 (1.8)
Controls	127 (12.7)	136 (0.14)	17 (1.7)	0 (0)	7 (0.7)
aPR [95% CI]	**1.59 [1.29–1.94]**	**1.72 [1.39–2.13]**	**3.45 [1.99–6.01]**	-	2.07 [0.88–4.85]
Cervical spine					
Retired Olympians	83 (5.6)	85 (0.06)	8 (0.5)	0 (0)	0 (0)
Control	33 (3.3)	34 (0.03)	5 (0.5)	0 (0)	0 (0)
aPR [95% CI]	**1.67 [1.10–2.54]**	**1.66 [1.08–2.54]**	1.06 [0.35–3.21]	-	-
Lumbar spine					
Retired Olympians	315 (21.2)	359 (0.24)	52 (3.5)	1 (0.1)	1 (0.1)
Controls	126 (12.6)	130 (0.13)	14 (1.4)	0 (0)	0 (0)
aPR [95% CI]	**1.66 [1.36–2.03]**	**1.85 [1.50–2.27]**	**2.33 [1.26–4.33]**	-	-
Hip					
Retired Olympians	91 (6.1)	100 (0.07)	46 (3.1)	19 (1.3)	2 (0.1)
Controls	42 (4.2)	45 (0.05)	19 (1.9)	11 (1.1)	0 (0)
aPR [95% CI]	**1.81 [1.21–2.72]**	**1.88 [1.23–2.86]**	1.56 [0.89–2.75]	1.05 [0.46–2.39]	-
Knee					
Retired Olympians	399 (26.8)	539 (0.36)	197 (13.2)	15 (1.0)	77 (5.2)
Controls	223 (22.3)	258 (0.26)	81 (8.1)	3 (0.3)	32 (3.2)
aPR [95% CI]	**1.24 [1.06–1.44]**	**1.46 [1.24–1.72]**	**1.65 [1.28–2.14]**	**4.66 [1.08–20.18]**	**1.73 [1.12–2.64]**
Ankle					
Retired Olympians	190 (12.8)	204 (0.14)	41 (2.8)	0 (0)	15 (1.0)
Controls	160 (16.0)	182 (0.18)	23 (2.3)	0 (0)	10 (1.0)
aPR [95% CI]	**0.80 [0.65–0.98]**	**0.76 [0.65–0.94]**	1.15 [0.68–1.93]	-	0.92 [0.41–2.08]

Reference group = controls; bold text = statistically significant; percentages rounded to one decimal place

a*PR* adjusted prevalence ratio (adjusted for age and BMI), *CI* confidence interval

The lumbar spine, followed by the knee were the most frequently reported sites for ongoing stiffness and pain for both retired Olympians and controls (Table 4), with low back pain more prevalent in retired Olympians. The knee, lumbar spine and hip were the most frequently affected joints in terms of OA diagnoses in both retired Olympians and controls, with OA prevalence rates higher for the cervical spine (aPR 1.85, 95% CI 1.01–3.39) and hip (aPR 1.82, 95% CI 1.08–3.06) in retired Olympians.

**Table 4:** Prevalence of current stiffness, pain and OA, by joint, in retired female Olympians *versus* female general population controls

	Stiffness ≥ 1 month, *n* (%)	Pain ≥ 1 month, *n* (%)	OA, *n* (%)
Shoulder			
Retired Olympians	124 (8.3)	122 (8.2)	37 (2.5)
Controls	86 (8.6)	81 (8.1)	11 (1.1)
aPR [95% CI]^†^	0.99 [0.75–1.31]	1.01 [0.76–1.35]	**2.05 [1.02–4.15]**
aPR [95% CI]*	0.82 [0.63–1.08]	0.82 [0.62–1.07]	1.80 [0.90–3.59]
Cervical spine			
Retired Olympians	175 (11.8)	130 (8.7)	50 (3.4)
Control	78 (7.8)	67 (6.7)	15 (1.5)
aPR [95% CI]^†^	**1.51 [1.15–1.99]**	1.31 [0.97–1.78]	**2.21 [1.20–4.06]**
aPR [95% CI]*	**1.42 [1.08–1.86]**	1.22 [0.90–1.65]	**1.85 [1.01–3.39]**
Lumbar spine			
Retired Olympians	367 (24.7)	285 (19.2)	100 (6.7)
Controls	149 (14.9)	114 (11.4)	47 (4.7)
aPR [95% CI]^†^	**1.66 [1.38–1.99]**	**1.67 [1.35–2.06]**	1.25 [0.88–1.79]
aPR [95% CI]*	**1.41 [1.18–1.67]**	**1.41 [1.15–1.73]**	1.04 [0.74–1.47]
Hip			
Retired Olympians	132 (8.9)	102 (6.9)	52 (3.5)
Controls	68 (6.8)	52 (5.2)	18 (1.8)
aPR [95% CI]^†^	**1.44 [1.07–1.94]**	**1.42 [1.01–2.00]**	**1.88 [1.06–3.34]**
aPR [95% CI]*	1.31 [0.98–1.76]	1.27 [0.91–1.77]	**1.82 [1.08–3.06]**
Knee			
Retired Olympians	229 (15.4)	198 (13.3)	139 (9.3)
Controls	116 (11.6)	98 (9.8)	62 (6.2)
aPR [95% CI]^†^	**1.33 [1.07–1.65]**	**1.35 [1.06–1.71]**	**1.46 [1.07–1.98]**
aPR [95% CI]*	1.18 [0.96–1.45]	1.19 [0.95–1.50]	1.25 [0.93–1.67]
Ankle			
Retired Olympians	70 (4.7)	47 (3.2)	19 (1.3)
Controls	56 (5.6)	40 (4.0)	8 (0.8)
aPR [95% CI]^†^	0.83 [0.58–1.18]	0.77 [0.50–1.19]	1.76 [0.73–4.23]
aPR [95% CI]*	0.91 [0.64–1.29]	0.85 [0.55–1.30]	1.92 [0.81–4.56]

Reference group = controls; bold text = statistically significant; percentages rounded to one decimal place

*aPR* adjusted prevalence ratio, *CI* confidence interval, *OA* osteoarthritis

^†^Adjusted for age and BMI

^*^Adjusted for age, BMI and previous significant injury in respective joint (yes/no)

### General Health

Retired Olympians and controls reported a similar prevalence of current disease states and medication use (Table 5). Comparison of disease states during the career of Olympians is presented in Supplementary Appendix 8 (see ESM). Increased prevalence rates from career to retirement of rheumatoid arthritis, osteoporosis, diabetes, hypertension and ‘other’ were evident in Olympians. Conversely, the self-reported prevalence of asthma significantly lowered post-career and depression also differed—increasing from in-career to during the retirement transition and then decreasing again in retirement (Supplementary Appendix 8, ESM).

**Table 5:** Overall self-reported prevalence of disease states, medication use, anxiety, depression, alcohol consumption and tobacco use in retired female Olympians versus general population controls

	Retired Olympians	Controls	aPR [95% CI]
Prevalence of current disease states, *n* (%)	*n* = 1488	*n* = 998	
Rheumatoid arthritis	28 (1.9)	13 (1.3)	1.61 [0.78–3.26]
Osteoporosis	41 (2.8)	31 (3.1)	0.81 [0.48–1.37]
Diabetes	13 (0.9)	4 (0.4)	1.95 [0.61–6.26]
Asthma	118 (7.9)	91 (9.1)	0.98 [0.74–1.30]
Epilepsy	5 (0.3)	1 (0.1)	1.99 [0.20–20.0]
Gout	6 (0.4)	2 (0.2)	1.28 [0.23–7.03]
Hypertension	60 (4.0)	34 (3.4)	1.34 [0.85–2.10]
Depression	92 (6.2)	82 (8.2)	0.84 [0.62–1.14]
Heart disease	9 (0.6)	4 (0.4)	0.96 [0.28–3.35]
Other heart issues	50 (3.4)	25 (2.5)	1.24 [0.75–2.07]
Lung disease	5 (0.3)	2 (0.2)	0.96 [0.15–5.96]
Other	144 (9.7)	111 (11.1)	0.88 [0.69–1.12]
Current medication use			
Overall prevalence, *n* (%)	281 (18.9)	229 (23.0)	0.86 [0.73–1.02]
Anti-inflammatories	194 (13.0)	112 (11.2)	1.17 [0.93–1.48]
Pain medication	126 (8.5)	96 (9.6)	0.91 [0.70–1.20]
HADS^‡^	*n* = 1195	*n* = 804	
Anxiety score ≥ 11, *n* (%)	139 (11.6)	120 (14.9)	0.82 [0.65–1.04]
Depression score ≥ 11, *n* (%)	40 (3.4)	22 (2.7)	1.27 [0.75–2.17]
Alcohol consumption, *n* (%)	*n* = 1488	*n* = 998	
Current drinker	912 (61.3)	663 (66.4)	**0.91 [0.86–0.97]**
Previous drinker	195 (13.1)	118 (11.8)	1.12 [0.89–1.41]
Never drank alcohol	159 (10.7)	73 (7.31)	**1.53 [1.15–2.02]**
Tobacco use, *n* (%)			
Current tobacco user	21 (1.4)	17 (1.7)	0.87 [0.44–1.72]
Previous tobacco user	87 (5.9)	144 (14.4)	**0.40 [0.30–0.52]**
Never tobacco user	1158 (77.8)	696 (69.7)	**1.11 [1.06–1.17]**

Reference group = controls; bold text = statistically significant; percentages rounded to one decimal place

Tobacco use: cigarette, cigar or pipe use

*aPR* adjusted prevalence ratio (adjusted for age and BMI), *CI* confidence interval, *HADS* Hospital Anxiety and Depression Scale

^‡^Missing values due to incomplete responses (retired Olympians, *n* = 293, controls *n* = 194)

No difference was observed in above-threshold rates of depression or anxiety when using the HADS tool. Internal consistency of both subscales was acceptable to good (Anxiety: McDonald’s *ω* = 0.83; Depression: McDonald’s *ω* = 0.78). Retired Olympians had slightly higher scores on the HADS depression subscale (median 3.0 vs 2.0, *p* < 0.05), while HADS anxiety subscale scores were similar between groups (median 5.0 vs 5.0, *p* > 0.05). EQ-5D-5L domains were rated similarly between groups with a difference only identified in the domain of anxiety/depression. When adjusting for current pain, a greater prevalence of retired Olympians report ‘any problems’ in this domain compared with controls (aPR 1.15, 95% CI 1.02–1.29).

### Sport, Exercise and Physical Activity

Fewer retired Olympians reported engaging in any sport, exercise or leisure PA weekly (aPR 0.95, 95% CI 0.92–0.98) compared with controls (Table 6). Current musculoskeletal pain (any joint) did not explain the differences in time spent participating in sport, exercise and leisure PA in the sub-group of retired Olympians and controls that report engaging in such activities (Supplementary Appendix 9, ESM). The most frequent forms of sport, exercise and leisure PA are detailed in Supplementary Appendix 10 in the ESM.

**Table 6:** Health-related quality of life and current sport participation, physical activity and exercise in retired female Olympians versus general population controls

	Retired Olympians	Controls	Adjusted PR^†^ [95% CI]	Adjusted PR* [95% CI]
Health-related quality of life (EQ-5D-5L), *n* (%)^‡^	*n* = 1245	*n* = 837		
Mobility				
No problems	1055 (84.7)	684 (81.7)	**1.04 [1.00–1.09]**	1.04 [0.99–1.09]
Any problem	190 (15.3)	153 (18.3)	**0.81 [0.66–0.99]**	0.82 [0.67–1.00]
Self-care				
No problems	1211 (97.3)	811 (96.9)	1.00 [0.99–1.02]	1.00 [0.99–1.02]
Any problem	34 (2.7)	26 (3.1)	0.85 [0.50–1.46]	0.87 [0.51–1.49]
Usual activities				
No problems	1067 (85.7)	695 (83.0)	1.04 [0.99–1.08]	1.04 [0.99–1.08]
Any problem	178 (14.3)	142 (17.0)	**0.80 [0.65–0.99]**	0.81 [0.66–1.00]
Pain/discomfort				
No problems	500 (40.2)	298 (35.6)	1.12 [0.99–1.26]	1.12 [0.99–1.27]
Any problem	745 (59.8)	539 (64.4)	0.93 [0.87–1.00]	0.93 [0.87–1.00]
Anxiety/depression				
No problems	754 (60.6)	543 (64.9)	**0.92 [0.86–0.99]**	**0.92 [0.86–0.99]**
Any problem	491 (39.4)	294 (35.1)	**1.14 [1.01–1.29]**	**1.15 [1.02–1.29]**
EQ VAS, median (IQR)	88 (80–95)	89 (80–95)		
Activity	*n* = 1488	*n* = 998		
Sport participation				
All sport	392 (26.3)	347 (34.8)	**0.76 [0.67–0.86]**	**0.75 [0.66–0.85]**
International level	137 (9.21)	148 (14.8)	**0.59 [0.47–0.74]**	**0.59 [0.47–0.74]**
National level	191 (12.8)	257 (25.8)	**0.51 [0.43–0.61]**	**0.50 [0.42–0.59]**
Recreational	559 (37.6)	660 (66.1)	**0.57 [0.52–0.61]**	**0.55 [0.51–0.60]**
Weekly duration (hours) median [IQR]	10 [5–18]	14 [7–23]		
Exercise				
Weekly prevalence, *n* (%)	1102 (74.1)	731 (73.3)	1.01 [0.97–1.06]	0.99 [0.94–1.04]
Weekly duration (hours) for those that participate in exercise, median [IQR]	6 [3–9]	5 [3–8]		
Leisure physical activity				
Weekly prevalence, *n* (%)	1133 (76.1)	741 (74.3)	**0.93 [0.89–0.97]**	**0.91 [0.87–0.95]**
Weekly duration (hours) median [IQR]	5 [3–10]	6 [3–10]		

Reference group = controls; bold text = statistically significant; weekly hours = for those that participate in PA; percentages rounded to one decimal place

aPR adjusted prevalence ratio, *CI* confidence interval, *EQ VAS* EuroQol Visual Analog Scale, *IQR* interquartile range

^†^Adjusted for age and BMI

^*^Adjusted for age, BMI and current **pain** in any joint (yes/no)

^‡^Missing values due to incomplete responses (retired Olympians, *n* = 243, controls *n* = 161)

## Discussion

This study aimed to address key gaps related to our understanding of the health of retired female Olympians from a global, multi-sport perspective. The main findings were (i) current joint health (pain, stiffness, OA) at the cervical spine, lumbar spine, hip and knee was poorer for retired female Olympians compared with female controls; (ii) health-related quality of life was rated similarly, with no difference in rates of current disease states; however, cases of diabetes, hypertension, rheumatoid arthritis and osteoporosis increased for Olympians from their careers to retirement; (iii) retired female Olympians self-reported experiencing a spike in depression as they transitioned through retirement, but this appears to have reduced over time, as self-reported depression rates were lower in retirement compared with during their Olympic career. Current levels of anxiety and depression in retirement were similar to controls; and (iv) the prevalence of any weekly participation in sport and leisure PA was lower in retired female Olympians compared with female general population controls; however, participation in any regular exercise was similar.

### Current Musculoskeletal Health

Musculoskeletal issues such as pain, stiffness and OA were more prevalent in retired female Olympians across five joints (shoulder, cervical spine, lumbar spine, hip and knee), although the pattern varied by outcome, joint and adjustment model. While the knee was the most prevalent site of previous significant injury among retired Olympians, the lumbar spine was the most prevalent site of current stiffness and pain, with each affecting 25% and 20% of retired female Olympians for most days of the last month, respectively. Our finding coincides with prior reports of a high prevalence of low back pain (LBP) among retired female Team GB Olympians [40] and retired female Canadian rugby players and rowers [24]. Previous LBP or injury appears to contribute to this presentation in retirement but does not fully explain it. When adjusting for previous significant lumbar injury, LBP and stiffness remain more prevalent in retired Olympians than in controls. Higher training volumes, periods of load increase and accumulated exposure over years are both characteristics of Olympic careers *and* risk factors for low back pain (LBP) in currently active athletic populations [41]. Insufficient management of LBP during an athletic career—such as care rooted in structural pathology [42, 43], or ongoing overuse through return to or continuing participation without full symptom resolution [19, 44]—may contribute to a high risk of chronic LBP in retirement for female Olympians, potentially through central sensitisation and nociplastic pain pathways [45]. These factors may represent important targets for mitigating LBP in active and retired female athletes; however, critical gaps specific to spinal pain prevention in female/woman/girl athletes remain, limiting tangible impact for female athletes experiencing persistent pain until these gaps are adequately addressed [46]. Additionally, qualitative exploration of persistent LBP and stiffness in retired female athletes is warranted to better understand the lived experiences and long-term quality-of-life impacts beyond athletic performance.

### Osteoarthritis

Diagnoses of OA were reported by one in every five retired female Olympians (20.4% versus 13.1% in controls), with the knee the most frequent site of OA diagnosis for both groups (9.3% versus 6.2%). Overall, knee OA was 46% more prevalent in retired Olympians, however, the difference was no longer significant after adjusting for previous knee injury. This indicates that Olympians were no more likely to suffer from knee OA than the general population if they had not previously experienced a significant knee injury. This finding aligns with the existing literature, which consistently recognises prior knee injury as a risk factor for the development of knee OA in both the general and, in particular, retired athletic populations [15, 29, 47]. With the knee cited as the most frequently injured location during the most recent Olympic Games across all sports [48, 49], our findings continue to reinforce that primary prevention of knee injury occurrence may be the most effective strategy for promoting long-term knee health in retired female Olympians [50].

While differences in some knee outcomes were observed between groups, the magnitude of these differences to general population controls was comparably lower than other joints. The greatest between-group differences in OA diagnoses were observed at the shoulder and cervical spine. The prevalence of each was more than double for the retired Olympians compared with controls yet affected < 2.5% and 3.4% of the entire retired Olympian cohort for both shoulder and cervical spine OA, respectively. Shoulder OA is somewhat understudied, but repetitive overhead sports, recurrent instability and trauma may be risk factors for early onset [51]. When PRs were adjusted for previous injury, the rates of OA at the shoulder were no longer higher for Olympians, meaning that prior significant shoulder injury largely explained the difference in shoulder OA relative to controls. This again emphasises the need to focus on primary prevention of shoulder injury with consideration to shoulder health in retirement [52]. Future research should also explore the specific profile of those who develop shoulder OA following significant injury versus those who do not. Similarly, sustained exposure to contact sports or repetitive forces may increase the risk of cervical spine degeneration [53] and provide a partial rationale for the increased prevalence of OA at these sites in retired Olympians, considering this difference remains even when prior cervical spine injury is accounted for.

### General Health

Overall, retired Olympians’ median self-rating of health was high (88 out of a possible 100), just one point lower than controls. Thus, despite differences in their injury history and current musculoskeletal health profile, retired Olympians perceive their health as positively as the female general population. Retired elite female rugby players and rowers in Canada also rated their current health highly, with 82% of that sample rating their health as ‘above average’ or ‘excellent’ [24], while 70% of retired female professional soccer players described their current health as ‘good’ or ‘very good’ [47]. Interestingly, fewer retired Olympians in the present study reported mobility or usual activity problems compared with controls, despite experiencing a higher prevalence of current musculoskeletal issues across various joints. The apparent disconnect between musculoskeletal complaints and other general health measures may be three-fold. First, athletes may exhibit greater pain tolerance and process pain differently than the general population [54, 55]. Second, elite athletes develop and utilise psychological factors through their experience of career stressors that likely are maintained through to retirement. For example, optimism—a characteristic of successful elite athletes [56]—is linked to lower pain sensitivity through the reduction of pain catastrophising [57]. Prolonged exposure to elite sporting social environments, in which pain and injury are anticipated and solicitous responses may be less common, may also affect the operant conditioning of Olympians’ responses to painful stimuli in later life [57]. Third, athletes may recognise and intuitively understand the importance of movement and physical activity as a therapeutic modality [58] and use this to help moderate and manage their symptoms.

That said, the sport, exercise and leisure PA behaviours of retired female Olympians and general population controls differed. This difference in weekly participation and duration cannot be attributed to pain acting as a barrier as we earlier proposed. Similar proportions of both groups engage in exercise weekly (74% vs 73%) with retired Olympians spending slightly more time (~ 30 min more per week) exercising. This is despite higher rates of joint stiffness, pain and OA, supporting the idea that athletes may be using exercise to moderate their symptoms. In contrast, 24–25% fewer retired Olympians reported currently participating in organised sport and those who did spent 3–3.5 h less on sporting activities per week than controls. The reasons for these discrepancies are unclear, but our analysis *does not* indicate that current pain was an influential factor, that is, when adjusting for the presence of current pain, the differences in sport participation rates remained unchanged. It is possible that release from the strict scheduling of elite and professional sport has manifested in a less formal relationship with sport in retirement, coupled with increased opportunity for the diversification of their hobbies and interests [59]. Motherhood, too, represents an additional gendered consideration for retired female Olympians, with caregiving responsibilities limiting time available for consistent engagement in formalised sport [60]. With respect to differences in PA, it is possible that retired Olympians self-imposed stricter standards when assessing and reporting their level of PA—perhaps unconsciously comparing their current PA to the far more demanding physical requirements during their sporting careers—resulting in lower reported prevalence rates. Further research should explore how these and other barriers and facilitators influence the continuation of sport, exercise and leisure PA in retired Olympians as the maintenance of these behaviours as individuals age is essential to mitigating a wide range of negative health outcomes [25].

At this cross-sectional timepoint, however, retired Olympians did not illustrate any clear differences in prevalent disease states compared with controls—suggesting that some superior health benefits accrued during their Olympic careers [61] may not be maintained long term. Both groups reported similar prevalence of diabetes and hypertension, which conflicts with studies of retired female GB Olympians [23] and retired elite female rugby players and rowers [24], where fewer former athletes reported such conditions. Additionally, increases in both diabetes and hypertension were evident from career to retirement for Olympians. This may suggest the loss of some health benefits accrued during their Olympic career through changes in lifestyle following this transition, such as reductions in physical activity, or possibly the consequential impact of former and/or continued dietary behaviours such as high caloric intake to support energy expenditure [5]. However, due to the paucity of female-specific cardiovascular health data, current beliefs are largely derived from male-only datasets [5].

In contrast, we observed some positive trends for the mental health trajectory of female Olympians in retirement. The prevalence of self-reported depression decreased from 9.2% during their careers and 12.8% during the transition period to 6.2% in retirement. Extant literature has documented how this transitionary period is a vulnerable time for elite athletes—with stronger athletic identity and an involuntary retirement correlated with the quality of career transition [62]. In addition, a recent systematic review also considered female sex/gender as a possible correlate with negative mental health at this time based upon a narrative summary of ten studies [63]. Despite this apparent spike across the transition into retirement, our results indicate that current rates of depression and anxiety in female Olympians return to a similar rate in retirement when compared with general population controls. This aligns with findings of a recent meta-analysis [6] investigating the mental health of elite athletes whereby symptoms of anxiety and depression were more prevalent in current (34%) versus former elite athletes (26%) [6]. While this previous research also indicates mental health may improve following retirement from elite sport given potentially lower exposure to adverse life events and sport-specific stressors [64, 65], our findings extend this evidence. The current study demonstrates a similar pattern among retired elite female athletes (specifically Olympians), with self-reported rates of depression declining from the in-career to post-career period, although the proportion of affected individuals was lower among the current female cohort overall. In contrast, a second meta-analysis of retired elite athletes reported *greater* proportions of mental health problems in retired athletes compared with the general population [22], a finding which was not observed in the current study or in other retired female elite athlete samples [23, 24]. The reason for these differences (i.e., overall proportion of anxiety and depression [6]; conflicting comparisons with the general population [22]) between these findings and our study may largely be due to the disproportionately low representation of female athletes in these aforementioned meta-analyses with just 2.6% (*n* = 165) and 2.0% (*n* = 492) of retired female elite athletes comprising their overall participant samples [6, 22]—much smaller than the 1488 retired female Olympians surveyed here. Our and other female-specific study findings may suggest that retired elite female athletes experience more favourable mental health outcomes and psychosocial adjustment following athletic retirement compared with their male counterparts; however further investigations are needed to further clarify these findings and to establish mechanisms.

### Strengths and Limitations

This is the first, and largest, global female-specific exploration of retired Olympian musculoskeletal and general health, including comparison with a cohort of similar aged female general population controls. In an effort to recruit a globally diverse sample, the survey was made available in eight languages supporting responses from athletes that represented 132 NOCs at both the Winter and Summer Games. However, 56% of the retired female Olympian cohort was represented by the United States (17%), Great Britain (15%), Australia (13%) and Canada (11%). These NOCs often have the largest teams participating at Olympic Games and therefore, unavoidably have a greater retired population from which to draw, limiting the generalisability of our findings. The overall size of the global retired female Olympian population is not accurately known, nor was it possible to measure the reach of this survey’s distribution and calculate a response rate. Additionally, retired Summer Olympians comprise the majority of our recruited sample, limiting generalisability of our findings to retired Winter Olympians. However, the proportion of female Summer and Winter Olympians does sit within the region of an 80:20 ratio as per indications from the three most recent Summer and Winter Games participation rates and so our cohort does likely reflect the proportional breakdown of the wider retired female Olympian population. The general population control cohort shared a similar geographical spread as the Olympian group—with 69.9% of the control cohort residing in Australia, Canada, Great Britain or the United States at the time of data collection. Although efforts were made to recruit an ethnically diverse sample, representation remained limited, with white participants comprising 78.8% of the retired Olympian cohort and 90% of the control cohort. While this study aids in advancing sex/gender equality through female-specific research, it does not additionally account for the intersection of race, culture and economics. Future research must take deliberate action to facilitate geographic and ethnic diversity in participant samples to better serve female athletes globally. A further potential limitation is related to the recruitment strategy for the general population control group. Specifically, the two strands of this strategy that likely identified individuals adjacent to physically active people (retired Olympians, sports medicine researchers) which may have influenced this sample to be more physically active (and therefore healthier) than is true for the general population. Hence, it is possible that the magnitude of difference between groups may have been underestimated for outcomes known to be associated with physical activity, such as significant musculoskeletal injury history, some disease states and mental health status.

This cross-sectional study is likely, in parts, to be affected by recall bias. To mitigate against this, ‘significant injury’ was defined as ‘any injury that caused pain and discomfort for most days for at least one month’. This operational definition was employed to capture data related to the injuries participants would most likely recall. By employing this method, we acknowledge that less severe injuries are unlikely to have been captured. This manuscript provided detailed analysis of six anatomical regions identified a priori as the most commonly affected across both groups. While this approach supports clinical relevance, differences in less frequently affected regions were not explored.

Measurement invariance and formal analysis comparing the characteristics of participants with complete and incomplete questionnaire data was not conducted for this study. While we have utilised tools validated across different languages and populations, this does not infer or equate to measurement invariance. Therefore, we cannot assume that the adopted instruments captured constructs consistently, thereby limiting analysis of between-group comparisons. We also adopted ‘complete-case analysis’ and assumed data were missing completely at random—however, this was not formally assessed. This may introduce selection bias as those that returned complete instrument responses for the HADS and EQ-5D-5L tools may have differed systematically from those that returned incomplete responses. For the HADS mental health assessment tool, a cut-off score of 11 was applied, which likely identified only those with more severe cases of depression. This was done to minimise false positives, maximise identification of symptom severity [35] and to compare with the only other study that uses a threshold HADS score in a retired elite athlete population [66]. This may explain the discrepancy in depression results, as this high clinical threshold was applied when scoring the HADS depression subscale, in contrast to participants’ subjective perception of ‘suffering with depression’ that led to their inclusion in self-reported depression rates. In addition, our collapsed categorical responses using the EQ-5D-5L measure creates the opposite issue, as our approach aggregates the spectrum of symptom severity into one category. Relating our findings to other literature is limited in places due to disproportionate studies of retired male athletes and a lack of comparable datasets in current or retired female elite athletes. Finally, our findings are limited in that they do not address any female-specific health domains (e.g., reproductive/endocrinological, breast, pelvic floor health) which may be influential in some of the outcomes described, and indeed warrant comprehensive exploration as distinct topics of investigation in this cohort.

## Conclusions

This study provides important new insights to address gaps in our understanding of retired female Olympian health. The self-reported musculoskeletal health of retired female Olympians is poorer than female general population controls, with an increased prevalence of pain, stiffness and/or OA at the shoulder, cervical spine, lumbar spine, hip and knee—only some of which can be explained by prior significant injury. Retired Olympians rate their health-related quality of life and score their mental health similarly to controls, raising suggestions of differing pain tolerance, learned coping mechanisms and/or increased awareness of the therapeutic use of exercise to manage their symptoms. Furthermore, retired female Olympians report differing—mostly lower—levels of engagement in sport, exercise and PA behaviours; however, analysis failed to identify persistent pain as a barrier.

Future research should look to broaden our understanding of health domains that remain heavily understudied in retired female athletes, such as sleep, cognitive and cardiovascular health. Critically, research should also encompass female-specific health domains such as reproductive/endocrinological health, bone health, pelvic floor and breast health, as the long-term implications of sustained, intense sport participation on these body systems is unknown. Focused efforts to understand and support the health of active and retired female Olympians is imperative to protect and promote future generations of female athletes both in career and beyond the podium.

## Supplementary Information

Below is the link to the electronic supplementary material.

Supplementary file1 (DOCX 242 KB)

## Declarations

### Availability of data and material

The data that support the findings of this study are not openly available due to reasons of sensitivity and are available from the corresponding author upon reasonable request.
