# Supplementary material for: The Legacy of Olympic Sport: A Cross-Sectional Comparison of the Musculoskeletal and General Health of 1488 Retired Female Summer and Winter Olympians and 998 Female General Population Controls
Source: Sports Med. 2026 Jul 23;56(12):3169–84. doi: 10.1007/s40279-026-02497-2 (PMC13759542; doi:10.1007/s40279-026-02497-2)
Supplement: Supplementary file 1 [file 40279_2026_2497_MOESM1_ESM.docx]

**The legacy of Olympic sport: A cross-sectional comparison of the musculoskeletal and general health of 1,488 retired female summer and winter Olympians and 998 female general population controls**

Lauren Guilfoyle^1,2^, Alan MacPherson^1,2^, Dale Cooper^3^, Kay M Crossley^4,5^, Amber T. Donaldson^6,7^, Niall Elliott^2^, Lars Engebretsen^8^, Suzanne Leclerc^9,10^, Eric G Post^6,7^, Craig Ranson^11^, Patrick Schamasch^12^, Malav Shroff^12^, Jenna M Schulz^13^, Torbjørn Soligard^14^, Jane S Thornton^14^, Mike Wilkinson^16,17^, Debbie Palmer^1,2^

1. Edinburgh Sports Medicine Research Centre, University of Edinburgh Institute for Sport Physical Education and Health Sciences, Edinburgh, UK
2. UK Collaborating Centre on Injury and Illness Prevention in Sport, University of Edinburgh, Edinburgh, UK
3. School of Allied Health Professions, Keele University, Keele, Staffordshire, UK
4. La Trobe Sport and Exercise Medicine Research Centre, La Trobe University, Australia
5. Australian IOC Research Centre, La Trobe University, Australia
6. Department of Sports Medicine, United States Olympic & Paralympic Committee,

Colorado Springs, Colorado, USA

7. US Coalition for the Prevention of Illness and Injury in Sport, Colorado Springs,

Colorado, USA

8. Department of Sports Medicine, Oslo Sports Trauma Research Center, Norwegian School of Sport Sciences, Oslo, Norway
9. Department of Family Medicine, University of Montreal, Montreal, Quebec, Canada
10. ReFORM IOC Research Centre for Prevention of Injury and Protection of Athlete Health
11. UK Sports Institute, Manchester, UK
12. Medical Committee, World Olympians Association, Lausanne Switzerland
13. Department of Physical Therapy, Faculty of Medicine, University of British Columbia, Vancouver, Canada
14. Health, Medicine and Science Department, International Olympic Committee, Lausanne, Switzerland
15. Department of Family Medicine, University of Montreal, Montreal, Quebec, Canada
16. Health and Wellbeing Department, Canadian Olympic Committee, Canada
17. Department of Sports Medicine, Faculty of Medicine, University of British Columbia, Vancouver, Canada

**Corresponding Author:** Dr Lauren Guilfoyle (Lauren.Guilfoyle@ed.ac.uk)

**Supplementary Material**

**Supplementary Appendix 1**

| Table. Overview of relevant survey content. | | | | |
| --- | --- | --- | --- | --- |
| Section | **Variable** | **Method of measurement/Question text** | **Difference within control survey** | **Origin or source** |
| Background | Age | Year of birth | N/D | A |
|  | Sex | Male, female, non-binary/third gender, prefer not to say | N/D | A |
|  | Ethnicity | Selected from a DDL | N/D | B |
|  | Country of residence | Selected from a DDL | N/D | A |
|  | Current height | Without shoes  Feet inches or centimetres  *Converted to centimetres* | N/D | A |
|  | Current weight | Without clothes  Stone pounds (lb) or kilograms  *Converted to kilograms* | N/D | A |
|  | Olympic career height | “During the peak of your sporting career”  Without shoes.  Feet inches or centimetres.  *Converted to centimetres.* | Question did not appear. | A |
|  | Olympic career weight | “During the peak of your sporting career”  Without clothes.  Stone pounds (lb) or kilograms.  *Converted to kilograms.* | Question did not appear. | A |
|  | Marital status | Current relationship status.  Available options: divorced, in a domestic partnership, married, separated, single but co-habiting with a significant other, single never married, widowed. | N/D | B |
|  | Education | Highest level completed.  Available options: did not finish school, school, college, university – undergraduate, university – postgraduate. | N/D | B |
|  | Employment status | Respondents were asked to report their current occupational status from a DDL: employed, unemployed or retired.  Unemployed or retired respondents could additionally select ‘for health reasons’ if this applied.  Employed respondents were additionally asked to select if their current employment was full-time or part-time; and if their current occupation involves moderate or strenuous activity (subjective). | N/D | B |
| Sport participation | Games participated at | Winter Olympic Games or Summer Olympic Games | Question did not appear. | A |
|  | NOC of representation | Selected from a DDL | Question did not appear. | A |
|  | Sport of Olympic representation | Selected from a DDL | Question did not appear. | A |
|  | International seasons | “How many seasons in total did you compete in Internationally during this time” - years selected. | Question did not appear. | A |
|  | Age at retirement from Olympic career | Year of retirement selected. *Post-hoc calculation to ascertain age at retirement (year of retirement – year of birth)* | Question did not appear. | A |
|  | Other representation | Repeated questions for change of NOC and/or sport representation. | Question did not appear. | N/A |
| Significant injury history | Significant injury prevalence | Respondents were asked to report incidence(s) of significant injuries that aligned with the provided definition “as any injury that caused pain and discomfort for most days for at least one month”. | N/D | C [1] |
|  | Injury location of each significant injury occurrence. | Selected from a DDL: abdomen, Achilles, tendon, ankle, elbow, face, finger, foot, forearm, groin, hand, head, hip, knee, lower back/lumbar spine, lower leg, neck/cervical spine, pelvis/sacrum/buttock, shoulder/clavicle, sternum/ribs, thigh, thumb, toe, upper arm, upper back/thoracic spine, wrist. | N/D | D [2] |
|  | Injury type | Selected from a DDL: arthritis/synovitis/bursitis, concussion, contusion/haematoma/bruising, dislocation/subluxation, fasciitis/aponeurosis injury, fracture, impingement, laceration/abrasion/skin lesion, lesion of meniscus or cartilage, ligamentous rupture, muscle cramps or spasm, nerve injury/spinal cord injury, other, other bone injuries, sprain, strain/muscle rupture/tear, stress fracture, tendinosis/tendinopathy, tendon rupture | N/D | D [2] |
|  | Injury cause | Multiple selection from DDL: overuse (gradual onset), overuse (sudden onset), non-contact trauma, recurrence of previous injury, contact with another athlete, contact with moving object, contact with stagnant object, violation of rules, field of play conditions, weather condition, equipment failure, other. | Within the sections ‘non-career injuries’ (retired Olympians) and ‘any significant career injuries (controls)’ - these responses (DDL) only displayed if the ‘place of injury’ selected was ‘sport’. | D [2] |
|  | Period of injury occurrence | *Injury reports were separated (for retired Olympians) in post-hoc analysis depending on where the injury report was inputted.*  Olympic career injury: reports inputted under “Olympic sport-related injury: Please indicate any/all **significant injuries that you suffered as a result of your Olympic career”.  Non-Olympic career injury: reports inputted under “Other significant injury history:  Please indicate (not including those incurred during your Olympic career) any/all other *significant injuries that you have suffered. This includes those incurred outside of sport.” | The retired Olympian survey version asked Olympians first to report any significant injury that occurred during their careers. The same set of questions – this time referring to any injuries that occurred after Olympic career retirement - was then posed.    In the control survey, respondents were asked to report any significant injury that has occurred to them. | A |
|  | History of joint surgery | “Have you ever had any spine or joint surgery, or replacement?”: Yes, No    If yes, respondents were asked to select the affected joint(s) and type(s) of surgery (DDL: arthroscopy, fusion, joint resurfacing, other, reconstruction i.e. ligament, total joint replacement). | N/D | C [1, 3], E |
| Current musculoskeletal health | Current pain or stiffness | “Spine or joint stiffness and/or pain - Please indicate whereby body joint you have had stiffness and/or pain for most days of the last month”: DDL of anatomical locations that permitted multiple selections. If a body joint was selected, the following questions were posed. | N/D | C [1, 3], E |
|  | Current stiffness | Do you experience stiffness (for most days of the last month): Yes, No | N/D | C [1] |
|  | Current pain | Do you currently experience pain (for most days of the last month) in this joint: Yes, No | N/D | C [1] |
|  | Current self-report physician-diagnosed OA | “Have you ever been diagnosed with osteoarthritis/degenerative joint disease in any of your joints by a medical professional”: Yes, No.    If yes, respondents were asked to select the affected joint(s). | N/D | C [1] |
| General health, quality of life, currently weekly sport, exercise and leisure physical activity | Self-reported disease states | “Disease states: Do you currently suffer from any of the following diseases. Select all that apply.    DDL: Rheumatoid Arthritis, Osteoporosis, Diabetes, Asthma, Epilepsy, Gout  High blood pressure, Depression, Heart disease, Other heart problems (e.g. arrhythmia), Lung disease, Other      Disease states: During your sporting career did you ever suffer any of these diseases. Select all that apply”    DDL: Rheumatoid Arthritis, Osteoporosis, Diabetes, Asthma, Epilepsy, Gout  High blood pressure, Depression – during career, Depression – on retirement, Heart disease, Other heart problems (e.g. arrhythmia), Lung disease, Other | The question related to disease states ‘during your sporting career’ did not appear. | B, C [1,3] |
|  | Medication use | “Medication use: Do you *currently regularly* take medication for any of these diseases. Select all that apply:”  Rheumatoid Arthritis, Osteoporosis, Diabetes, Asthma, Epilepsy, Gout  High blood pressure, Depression, Heart disease, Other heart problems (e.g. arrhythmia), Lung disease, Other    “Do you *currently regularly* take any of the following?”: Anti-inflammatories, Pain killers    “Medication use: *During your sporting career* did you regularly take medication for any of these diseases. Select all that apply”:  Rheumatoid Arthritis, Osteoporosis, Diabetes, Asthma, Epilepsy, Gout  High blood pressure, Depression, Heart disease, Other heart problems (e.g. arrhythmia), Lung disease, Other      *During your sporting career* did you ever regularly take any of the following?   Anti-inflammatories, Pain killers | The questions related to medication use ‘during your sporting career’ did not appear. | B |
|  | Anxiety | Hospital Anxiety and Depression Scale – Anxiety Subscale.  7-items  4-point Likert scale where responses are a variation of 0 = “not at all” and 3 = “most of the time” | N/D | F [4] |
|  | Depression | Hospital Anxiety and Depression Scale – Depression Subscale.  7-items  4-point Likert scale where responses are a variation of 0 = “not at all” and 3 = “most of the time”  A cut-off score of ≥11 (sensitivity: 0.64, specificity 0.87 [5]) was adopted to minimise false positives, and to be comparable to the only other study that uses a threshold HADS score in a retired elite athlete population [3]. | N/D |  |
|  | Alcohol consumption | “Alcohol Consumption: Do you drink?”  DDL: Yes; No, I do not currently drink but I have in the past; No, I have never drunk alcohol in my life | N/D | A |
|  | Tobacco use | “Tobacco: Do you currently smoke (cigarette, cigar or pipe)”  DDL: Yes; No – I am an ex-smoker; No – I am a non-smoker | N/D | A |
|  | Health-related quality of life | EuroQol EQ-5D-5L measure.  Participants are presented with statements that describe their current experience of five domains of health (mobility, self-care, usual activities, pain/discomfort, anxiety, depression). They are asked to select the response that best describes their current health: no problems, slight problems, moderate problems, severe problems, unable to/extreme problems    EQ-VAS: “This scale is numbers from 0 to 100. 100 means the best health you can imagine, 0 means the worst health you can imagine. Please click on the drop down menu to indicate how your health is TODAY”. Respondents select a number via sliding scale. | N/D | F [6, 7) |
| Sport, exercise and physical activity | Sport | “Other regular sporting training and competition exposure: We would also like to know if you have taken part in any other competitive sport(s) during, or since you retired from Olympic level competition”    Level of participation: international, national, recreational.    Hours per week: training hrs per week + competition hours per week | The question text posed to controls omitted any reference to during or since retirement from Olympic-level competition. The input fields were otherwise consistent. | A |
|  | Exercise and PA – introductory text | “Current exercise and physical activities: Not including the organised sporting activities you’ve already told us about previously. Please include as many different types of exercise and general physical activity you currently do on a weekly basis:” | N/D | A |
|  | Current exercise | “Current exercise (e.g. running, swimming, aerobics, gym)”    Type of activity selected from DDM: cycling, swimming, fitness classes, gym, hiking, pilates, running, swimming, yoga, other    Level of exercise (linked to activity): light, moderate, vigorous    Hours per week: selected from DDL of absolute numbers | N/D | A |
|  | Current physical activity | “Physical activity (e.g. walking, shopping, gardening, DIY)”  Type of physical activity selected from DDL: DIY, gardening, shopping, walking    Hours per week: selected from DDL of absolute numbers | N/D | A |
| DDL, drop down list; N/D, no difference; N/A, not applicable, DIY, “Do It Yourself”.  A, Developed de novo/pragmatic; B, Developed de novo via steering group and refined at pilot testing; C, Aligned with previous literature in a comparable cohort; D, Categorisation as per the IOC Consensus Statement; E, Aligned with studies undertaken within the Arthritis Research UK, Centre for Sport, Exercise and Osteoarthritis; F, Developed for and validated in a general population; [ ], Numbers in parentheses denote the source material – full reference list available at end of supplementary material; [1], Cooper et al., 2018; [2] Junge et al., 2008; [3], Fernandes et al., 2019; [4], Zigmond & Snaith 1983; [5], Olssøn et al., 2005; [6], Herdman et al., 2011; [7], Feng et al., 2021. | | | | |

**Supplementary Appendix 2**

**Figure. Participant flow diagram**

*(Note: HADS, Hospital Anxiety and Depression Scale; EQ5D5L, EuroQol health-related quality of life across five domains).*

**Supplementary Appendix 3**

| Table. National Olympic Committee of representation of retired female Olympians. | | | | | | | | | |
| --- | --- | --- | --- | --- | --- | --- | --- | --- | --- |
| NOC | **n** | **NOC** | **n** | **NOC** | **n** | **NOC** | **n** | **NOC** | **n** |
| Angola | 2 | Cook Islands | 3 | Hong-Kong | 4 | Netherlands | 19 | Slovenia | 16 |
| Argentina | 20 | Costa Rica | 4 | Hungary | 3 | Netherlands Antilles | 2 | South Africa | 17 |
| Armenia | 1 | Croatia | 5 | Iceland | 1 | New Zealand | 55 | Spain | 5 |
| Aruba | 2 | Cuba | 2 | India | 7 | Nigeria | 2 | Sri Lanka | 3 |
| Australia | 186 | Cyprus | 7 | Indonesia | 3 | Norway | 4 | Suriname | 2 |
| Austria | 11 | Czech Republic | 6 | Ireland | 7 | Pakistan | 2 | Sweden | 40 |
| Azerbaijan | 1 | Czechoslovakia | 5 | Israel | 4 | Palestine | 1 | Switzerland | 12 |
| Bahamas | 1 | Democratic Republic of the Congo | 1 | Italy | 6 | Panama | 2 | Thailand | 1 |
| Bahrain | 1 | Denmark | 7 | Jamaica | 7 | Papua New Guinea | 1 | The Former Yugoslav Republic of Macedonia | 1 |
| Barbados | 1 | Ecuador | 1 | Japan | 53 | People’s Republic of China | 1 | Togo | 1 |
| Belarus | 2 | Egypt | 6 | Jordan | 1 | Peru | 8 | Trinidad and Tobago | 1 |
| Belgium | 21 | El Salvador | 3 | Kenya | 2 | Philippines | 3 | Tunisia | 1 |
| Belize | 1 | Estonia | 2 | Latvia | 1 | Poland | 1 | Turkey | 1 |
| Bermuda | 3 | Federal Republic of Germany | 9 | Liberia | 1 | Portugal | 4 | Ukraine | 6 |
| Bhutan | 1 | Fiji | 1 | Lithuania | 4 | Puerto Rico | 5 | United Republic of Tanzania | 1 |
| Brazil | 18 | Finland | 2 | Madagascar | 1 | Republic of Korea | 5 | United States of America | 251 |
| Burundi | 1 | France | 18 | Malaysia | 8 | Romania | 4 | Uruguay | 1 |
| Cameroon | 1 | German Democratic Republic | 1 | Mali | 2 | Russia | 3 | USSR | 4 |
| Canada | 170 | Germany | 12 | Malta | 2 | Russian Federation | 1 | Venezuela | 10 |
| Cape Verde | 2 | Ghana | 2 | Mauritania | 1 | Rwanda | 1 | Virgin Islands, US | 3 |
| Cayman Islands | 2 | Great Britain | 218 | Mauritius | 1 | Saint Lucia | 1 | Yugoslavia | 2 |
| Chile | 1 | Greece | 21 | Mexico | 27 | Serbia | 1 | Zimbabwe | 1 |
| China | 4 | Guatemala | 5 | Morocco | 1 | Singapore | 8 | Missing | 14 |
| Chinese Taipei | 1 | Haiti | 1 | Namibia | 1 | Slovakia | 4 | *Total* | *1488* |
| Colombia | 7 | Honduras | 2 | Nepal | 1 | Slovenia | 16 |  |  |

**Supplementary Appendix 4**

| Table. Country of current residence of female general population controls. | | | | | |
| --- | --- | --- | --- | --- | --- |
| Argentina | 2 | Guatemala | 1 | Portugal | 6 |
| Australia | 73 | Hungary | 3 | Puerto Rico | 1 |
| Austria | 3 | Iceland | 2 | Republic of Korea | 1 |
| Belgium | 2 | India | 2 | Republic of Moldova | 1 |
| Brazil | 5 | Ireland | 21 | Russia | 5 |
| Cameroon | 1 | Israel | 1 | Serbia | 1 |
| Canada | 222 | Italy | 6 | Singapore | 5 |
| Cayman Islands | 1 | Jamaica | 1 | Slovenia | 1 |
| Chile | 1 | Japan | 8 | South Africa | 2 |
| Chinese Taipei | 2 | Kenya | 1 | Spain | 2 |
| Columbia | 1 | Latvia | 3 | Sri Lanka | 1 |
| Costa Rica | 1 | Lithuania | 2 | Swaziland | 1 |
| Croatia | 1 | Malaysia | 2 | Sweden | 13 |
| Cyprus | 2 | Mauritius | 1 | Switzerland | 58 |
| Denmark | 4 | Mexico | 6 | United Arab Emirates | 3 |
| Fiji | 1 | Netherlands | 7 | United States of America | 140 |
| France | 7 | New Zealand | 10 | Missing | 10 |
| Germany | 23 | Norway | 52 | *Total* | *998* |
| Great Britain | 263 | Peru | 1 |  |  |
| Greece | 1 | Philippines | 1 |  |  |
| Guam | 1 | Poland | 1 |  |  |

**Supplementary Appendix 5**

| Table. Olympic sport of representation of retired female Olympians. | | | | | |
| --- | --- | --- | --- | --- | --- |
| Summer (n) | | | | **Winter (n)** | |
| Archery | 15 | Hockey | 56 | Alpine skiing | 39 |
| Athletics | 204 | Judo | 32 | Biathlon | 15 |
| Badminton | 16 | Modern pentathlon | 4 | Bobsleigh | 12 |
| Basketball | 32 | Rowing | 139 | Cross country skiing | 17 |
| Beach volleyball | 12 | Rugby Sevens | 12 | Curling | 14 |
| Boxing | 2 | Sailing | 53 | Figure skating | 18 |
| Canoe slalom | 2 | Shooting | 22 | Freestyle skiing | 23 |
| Canoe sprint | 37 | Softball | 13 | Ice hockey | 13 |
| Cycling - mountain biking | 3 | Swimming | 238 | Long track speed skating | 17 |
| Cycling - road | 19 | Synchronised swimming | 67 | Luge | 13 |
| Cycling - track | 7 | Table tennis | 10 | Short track speed skating | 17 |
| Diving | 37 | Taekwondo | 21 | Skeleton | 6 |
| Equestrian | 4 | Tennis | 8 | Ski jumping | 4 |
| Fencing | 19 | Triathlon | 10 | Snowboarding | 14 |
| Football | 7 | Volleyball | 26 | Missing | 1 |
| Golf | 1 | Water polo | 13 | *Total* | *224* |
| Gymnastics - artistic | 55 | Weightlifting | 9 |  |  |
| Gymnastics - rhythmic | 29 | Wrestling | 5 |  |  |
| Gymnastics - trampoline | 1 | Missing | 7 |  |  |
| Handball | 17 | *Total* | *1264* |  |  |

**Supplementary Appendix 6**

| Table. Retired female Olympian and general population control ethnicity. | | |
| --- | --- | --- |
|  | **Retired Olympians**  **(n=1488)** | **Control**  **(n=998)** |
| Ethnicity, n (%) |  |  |
| White | 1162 (78.1) | 898 (90.0) |
| Asian other | 65 (4.4) | 19 (1.9) |
| Mixed White/other | 47 (3.2) | 14 (1.4) |
| Asian Chinese | 32 (2.2) | 19 (1.9) |
| Black other | 25 (1.7) | 1 (0.1) |
| Black Caribbean | 24 (1.6) | 1 (0.1) |
| Black African | 21 (1.4) | 3 (0.3) |
| Mixed White/Asian | 21 (1.4) | 11 (1.1) |
| Mixed White/Black Caribbean | 20 (1.3) | 6 (0.6) |
| Mixed White/Black African | 11 (0.7) | 5 (0.5) |
| Arabian | 10 (0.7) | 1 (0.1) |
| Asian Indian | 10 (0.7) | 4 (0.4) |
| Asian Pakistani | 2 (0.1) | 0 (0.0) |
| Any other ethnic group | 31 (2.1) | 16 (1.6) |
| *Missing* | *7 (0.5)* | *0 (0.0)* |

**Supplementary Appendix 7**

| Table. Retired female Olympian and general population control marital status. | | |
| --- | --- | --- |
|  | **Retired Olympians**  **(n=1488)** | **Control**  **(n=998)** |
| Marital status, n (%) |  |  |
| Divorced | 90 (6.0) | 55 (5.5) |
| Domestic partnership or civil union | 160 (10.8) | 99 (9.9) |
| Married | 741 (49.8) | 439 (44.0) |
| Separated | 29 (1.9) | 18 (1.8) |
| Single, but cohabiting with significant other | 153 (10.3) | 91 (9.1) |
| Single, never married | 290 (19.5) | 279 (28.0) |
| Widowed | 19 (1.3) | 16 (1.6) |
| *Missing* | *6 (>0.1)* | *1 (0.1)* |

**Supplementary Appendix 8**

| Table. Disease states of retired female Olympians (n=1488) in career and in retirement | | | |
| --- | --- | --- | --- |
| Prevalence of current disease states, n (%) | **During career** | **In retirement** | **PR [95%CI]** |
| Rheumatoid arthritis | 11 (0.74) | 28 (1.88) | **2.55 [1.27-5.09]** |
| Osteoporosis | 6 (0.40) | 41 (2.76) | **6.83 [2.91-16.0]** |
| Diabetes | 4 (0.27) | 13 (0.87) | **3.25 [1.06-9.95]** |
| Asthma | 175 (11.8) | 118 (7.93) | **0.67 [0.54-0.84]** |
| Epilepsy | 6 (0.40) | 5 (0.34) | 0.83 [0.25-2.72] |
| Gout | 2 (0.13) | 6 (0.40) | 3.0 [0.61-14.8] |
| Hypertension | 11 (0.74) | 60 (4.03) | **5.45 [2.88-10.3]** |
| Depression – during career | 137 (9.21) | 92 (6.18) | **0.67 [0.52-0.87]** |
| Depression - during retirement transition | 190 (12.8)* | | **0.48 [0.38-0.62]**** |
| Heart disease | 5 (0.34) | 9 (0.60) | 1.80 [0.60-5.36] |
| Other heart issues | 33 (2.22) | 50 (3.36) | 1.52 [0.98-2.34] |
| Lung disease | 7 (0.47) | 5 (0.34) | 0.71 [0.23-2.25] |
| Other | 64 (4.30) | 144 (9.68) | **2.25 [1.69-2.99]** |
| *, depression during career versus during retirement transition (PR: 1.39, 95% CI 1.13-1.71); **, depression during transition compared to in retirement; *PR,* prevalence ratio (unadjusted); *CI*, confidence interval; bold text = statistically significant; reference group = in-career. | | | |

**Supplementary Appendix 9**

| Table. Adjusted median differences for sport, exercise and physical activity durations (hours) of retired female Olympians and general population controls. | | | | |
| --- | --- | --- | --- | --- |
|  | **Median [IQR]** | | **aMD^1^ [95% CI]** | **aMD^2^ [95% CI]** |
| Duration (hours) | **Retired Olympian** | **Control** |  |  |
| Sport | 10 [5-18] | 14 [7-23] | -3.3 [-4.7 to -1.9] | -3.0 [-4.4 to -1.6] |
| Exercise | 6 [3-9] | 5 [3-8] | 0.55 [0.07 to 1.02] | 0.36 [-0.15 to 0.86] |
| Physical Activity | 5 [3-10] | 6 [3-10] | -0.31 [-0.78 to 0.16] | -0.32 [-0.81 to 0.18] |
| Note: figures relate to those who report actively engaging in respective activities rather than representative of the entire sample. *IQR*, interquartile range; *aMD*, adjusted median difference analysed using quantile (median) regression; *aMD^1^*, adjusted for age and BMI; *aMD^2^*, adjusted for age, BMI and current pain in any joint (yes/no); *CI*, confidence interval. | | | | |

**Supplementary Appendix 10**

| Table. Most frequently reported sports of retired female Olympians and general population controls at the time of data collection. | | |
| --- | --- | --- |
| Sport, n (%) | **Retired Olympians (n=1488)** | **Controls (n=998)** |
| Athletics | 85 (5.7) | 56 (5.6) |
| Swimming | 53 (3.6) | 33 (3.3) |
| Road cycling | 46 (3.1) | 26 (2.6) |
| Golf | 28 (1.8) | 15 (1.5) |
| Triathlon | 26 (1.7) | 20 (2.0) |
| Rowing | 25 (1.7) | 13 (1.3) |
| Mountain biking | 24 (1.6) | 13 (1.3) |
| Tennis | 19 (1.3) | 17 (1.7) |

| Table. Most frequently reported physical activity choices of retired female Olympians and general population controls at the time of data collection. | | |
| --- | --- | --- |
| Physical activity, n (%) | **Retired Olympians (n=1488)** | **Controls (n=998)** |
| Walking | 994 (66.8) | 740 (74.1) |
| Shopping | 380 (25.5) | 321 (32.2) |
| Gardening | 304 (20.4) | 172 (17.2) |
| DIY | 172 (11.6) | 106 (10.6) |

| Table. Most frequently reported exercise choices of retired female Olympians and general population controls at the time of data collection. | | |
| --- | --- | --- |
| Exercise, n (%) | **Retired Olympians (n=1488)** | **Controls (n=998)** |
| Running | 530 (35.6) | 304 (30.2) |
| Gym | 487 (32.7) | 313 (31.4) |
| Cycling | 301 (20.2) | 202 (20.2) |
| Fitness classes | 286 (19.2) | 201 (20.1) |
| Hiking | 190 (12.8) | 142 (14.2) |

**Supplementary Appendices References:**

1. Cooper DJ, Scammell BE, Batt ME, Palmer D. Factors associated with pain and osteoarthritis at the hip and knee in Great Britain’s Olympians: a cross-sectional study. Br J Sports Med. 2018;52(17):1101-8. doi: <https://doi.org/10.1136/bjsports-2017-098315>
2. Junge A, Engebretsen L, Alonso JM, Renström P, Mountjoy M, Aubry M, Dvorak J. Injury surveillance in multi-sport events: the International Olympic Committee approach. Br J Sports Med. 2008;42(6):413-21. doi: <https://doi.org/10.1136/bjsm.2008.046631>
3. Fernandes GS, Parekh SM, Moses J, Fuller CW, Scammell B, Batt ME, Zhang W, Doherty M. Depressive symptoms and the general health of retired professional footballers compared with the general population in the UK: a case–control study. BMJ Open. 2019;9(9):e030056. doi: <https://doi.org/10.1136/bmjopen-2019-030056>
4. Zigmond AS, Snaith RP. The hospital anxiety and depression scale. Acta Psychiatr. Scand. 1983;67(6):361-70. doi: <https://doi.org/10.1111/j.1600-0447.1983.tb09716.x>
5. Olssøn I, Mykletun A, Dahl AA. The Hospital Anxiety and Depression Rating Scale: a cross-sectional study of psychometrics and case finding abilities in general practice. BMC Psychiatry. 2005;5(1):46. <https://doi.org/10.1186/1471-244X-5-46>
6. Herdman M, Gudex C, Lloyd A, Janssen MF, Kind P, Parkin D, Bonsel G, Badia X. Development and preliminary testing of the new five-level version of EQ-5D (EQ-5D-5L). Qual Life Res. 2011;20(10):1727-36. doi: <https://doi.org/10.1007/s11136-011-9903-x>
7. Feng YS, Kohlmann T, Janssen MF, Buchholz I. Psychometric properties of the EQ-5D-5L: a systematic review of the literature. Qual Life Res. 2021;30(3):647-73. Doi: <https://doi.org/10.1007/s11136-020-02688-y>
